# Electrochemical Small-Angle X-ray Scattering for Potential-Dependent Structural Analysis of Redox Enzymes

**DOI:** 10.1021/acs.langmuir.4c03661

**Published:** 2024-12-31

**Authors:** Noya Loew, Chika Miura, Chiaki Sawahara, Saki Otobe, Taku Ogura, Yuichi Takasaki, Hikari Watanabe, Isao Shitanda, Masayuki Itagaki

**Affiliations:** † Department of Pure and Applied Chemistry, Faculty of Science and Technology, Tokyo University of Science, 2641 Yamazaki, Noda, Chiba 278-8510, Japan; ‡ Nikko Chemicals Co. Ltd., 3-24-3 Hasune, Itabashi-ku, Tokyo 174-0046, Japan; § Research Institute for Science and Technology, Tokyo University of Science, 2641 Yamazaki, Noda, Chiba 278-8510, Japan; ∥ Anton Paar Japan K.K., Riverside Sumida 1F, 1-19-9, Tsutsumi-dori, Sumida-ku, Tokyo 131-0034, Japan

## Abstract

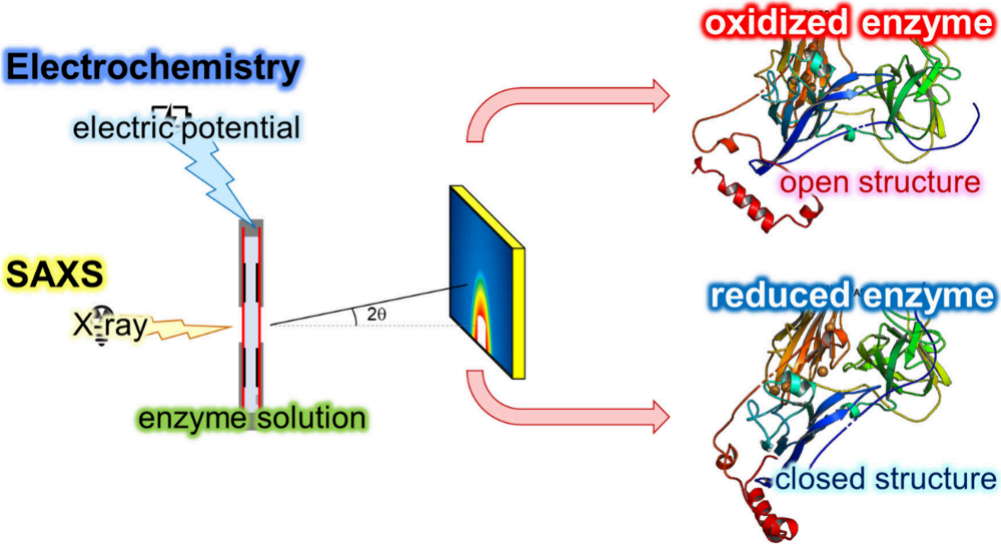

Various methods exist for exploring different aspects of these mechanisms. However, techniques for investigating structural differences between the reduced and oxidized forms of an enzyme are limited. Here, we propose electrochemical small-angle X-ray scattering (EC-SAXS) as a novel method for potential-dependent structural analysis of redox enzymes and redox-active proteins. While similar approaches have been employed previously in battery and fuel cell research, biological samples have not yet been analyzed using this technique. Using EC-SAXS, we elucidated the structures of oxidized and reduced bilirubin oxidase (BOD). The oxidized BOD favors an open state, enhancing accessibility to the active center, whereas the reduced BOD prefers a closed state. EC-SAXS not only broadens our understanding of redox enzymes but also offers insights that could aid in developing customized enzyme immobilization strategies. These strategies could considerably improve the performance of biosensors, biofuel cells, and other bioelectronics.

## Introduction

Redox-active enzymes are central to the ever-expanding field of biosensors, biofuel cells, and bioelectronics. A key component of these bioelectrochemical devices is the enzyme electrode, where redox enzymes are immobilized on the electrode surface. Among the various techniques for enzyme immobilization, covalent binding to the electrode surface is considered one of the most stable and rigid methods.^1−3^ However, covalent coupling may inactivate enzymes, which may occur because essential amino acid residues, necessary for maintaining structural integrity or facilitating the reaction mechanism, become nonfunctional, or because the necessary structural flexibility for enzyme activity is compromised by overly rigid immobilization.

The enzymes most commonly used in these devices, such as glucose oxidase (GOx),^4−7^ glucose dehydrogenase (GDH),^7−11^ lactate oxidase (LOx),^12−15^ and bilirubin oxidase (BOD),^16−22^ have been extensively characterized structurally, mechanistically, and electrochemically. The primary method for structural characterization of enzymes is X-ray crystallography, which yields high-resolution structure models. However, this method depends on crystalline samples and therefore restricts the conformational states that can be analyzed. At least two conformational states are of special interest for redox enzymes: the reduced and oxidized states. While some enzymes and redox proteins have been crystallized with oxidizing additives like ferricyanide,^23−25^ under aerobic versus anaerobic conditions,^19^ or in both oxidized and reduced forms,^26,27^ conducting these investigations remains challenging.

X-ray crystallography remains the gold standard for obtaining structural data on proteins. However, other techniques such as nuclear magnetic resonance (NMR) spectroscopy, cryogenic electron microscopy (cryo-EM), small-angle neutron scattering (SANS), and small-angle X-ray scattering (SAXS) provide unique information that X-ray crystallography cannot. NMR is particularly useful for studying protein structures in solution but is limited to small proteins and peptides.^28,29^ Conversely, cryo-EM, which involves imaging proteins embedded in vitreous ice, is generally suited for larger proteins.^30,31^ SANS and SAXS are probably the most versatile because they can accommodate a wide range of protein sizes and sample conditions.^32−34^ SAXS, in particular, has garnered increasing attention recently. Laboratory-based SAXS devices, available from various companies, allow researchers to conduct studies independently of synchrotron facilities.^33−38^ Typical SAXS measurements using laboratory-based devices take longer than those conducted at a synchrotron to compensate due to the low intensity of the X-ray beam—several minutes compared to just a fraction of a second. Nevertheless, the background scattering in modern devices is minimal, ensuring that signals are reliable. Furthermore, the reduced intensity of the X-ray beam in laboratory-based devices lowers the risk of radiation damage to the sample protein, facilitating repeated measurements of the same sample.

Key characteristics of the protein sample, such as maximal particle dimension (*D*_max_), radius of gyration (*R*_g_), molecular weight, and excluded particle volume, can be directly derived from SAXS scattering data.^37−39^ The analysis and modeling of protein structures from SAXS data have been remarkably enhanced by the development and introduction of specialized software, such as the ATSAS suite.^40^ This suite, designed specifically for SAXS analysis of biomolecules, includes tools for both *ab initio* and refinement modeling.^41−44^*Ab initio* modeling generates a bead model that represents the average shape of the sample protein,^41,42^ whereas refinement modeling adjusts an existing high-resolution protein structure with the experimental scattering pattern using normal-mode analysis.^43,44^

SAXS played a crucial role in discovering the interdomain flip-flop motion of cellobiose dehydrogenase (CDH) during its redox reaction.^45−47^ CDH comprises a haem-binding domain and a flavin-dependent domain, with the active center located in the latter. The haem-binding domain transfers electrons to an electron acceptor protein. Tan et al. found that CDH can exist in both open and closed states.^45^ The closed state is necessary for interdomain electron transfer from flavin to haem, whereas the open state allows access to the active center. Moreover, Harada et al. successfully visualized CDH motion using high-speed atomic force microscopy.^47^ These findings suggest that some redox enzymes undergo conformational changes during redox reactions. Information about such changes enriches our understanding of enzyme mechanics and could potentially aid in the development of specialized immobilization methods for bioelectrode fabrication. This leads to two pivotal questions: (1) Do smaller, single-domain redox enzymes also undergo conformational changes during redox reactions? (2) What are the general methods available for investigating such changes?

BOD serves as an example of a small, single-domain redox enzyme. Commonly used in oxygen-reducing electrodes, such as biocathodes in biofuel cells,^48−50^ BOD’s capability for direct electron transfer and high activity at neutral pHs have made it a preferred enzyme for such applications. As a member of the multicopper oxidase family, BOD is a monomeric enzyme with a molecular mass of 60 kDa and contains four copper atoms.^17,21^ Three of these copper atoms form a trinuclear cluster, known as the T2/T3 site, which consists of two type 2 and one type 3 coppers. The T2/T3 site is critical for binding dioxygen and reducing it to water. The fourth copper atom, located slightly further from the cluster, is classified as type 1. This T1 Cu plays a crucial role in transferring electrons to the electron acceptor, which can be either the substrate or an electrode.^17,51^ Proposed structural changes in the T2/T3 site upon reduction^52^ suggest that BOD is a promising candidate for a small redox enzyme that undergoes conformational changes during redox reactions.

Several studies have demonstrated the use of SAXS and SANS combined with electrochemistry in the fields of corrosion and batteries.^53−60^ Ingham et al. explored the CO_2_ corrosion of steel using *in situ* SAXS.^54,55^ They measured the surface of the sample/working electrode using grazing incidence SAXS (GI-SAXS) while applying a potential. GI-SAXS measures X-ray scattering at the surface of a solid sample and is thus a powerful method for investigating structural differences of the sample surface. Prehal et al. fabricated a battery cell that permits X-rays to pass through one electrode only and studied the conversion of molecular sulfur to lithium sulfide in lithium–sulfur batteries using operando SAXS.^56^ Similarly, they investigated the electrodeposition of iodine in nanoporous carbons.^57^ Their cell is commercially available as an SAXS battery cell from Anton Paar.^61^ Prabhu and Reipa investigated the structure of nanomaterial dispersion undergoing redox reaction using SANS.^58^ Similarly, Randle et al. used SANS to monitor electrochemical gelation.^60^

Another technique that combines electromagnetic radiation with electrochemistry is UV–vis spectroscopy, specifically in spectroelectrochemistry, which is often used to study redox enzymes. Mediated spectroelectrochemical titration, a variant of this method, involves oxidizing or reducing enzymes in solution with appropriate mediators by applying different potentials.^62−64^ The goal is to identify the potential at which the enzyme transitions between its oxidized and reduced forms, which exhibit distinct absorbance spectra. This transition potential corresponds to the redox potential of the enzyme. Several common redox enzymes, including BOD, have been characterized using this method.^62,63^

In this study, we drew insights from mediated spectroelectrochemical titration, *in situ* SAXS, and SAXS analysis of proteins to develop a novel method for the structural analysis of enzymes in their reduced and oxidized states, which we named electrochemical SAXS (EC-SAXS). By utilizing this innovative method, we investigated the structural differences between the oxidized and reduced forms of BOD and found that BOD forms an open or closed state depending on its redox state.

## Experimental Section

### Materials

Silver ink (SAP-40FL) was obtained from Sanwa Chemical Industrial, Japan. Carbon ink (JELCON CH-8) was purchased from Jujo Chemical, Japan. Resist ink (S-40 C518) was procured from Taiyo Ink, Japan. Polydimethylsiloxane (PDMS) base (KE-106) and the curing agent (CAT-RG) were obtained from Shin-Etsu Chemical, Japan. Low-temperature hardening epoxy resin adhesive 2-component type 2086N was purchased from ThreeBond Fine Chemical Co. Ltd., Japan. Conductive copper adhesive tape No. 8315 was provided by Teraoka Seisakusho Co., Ltd., Japan. Bilirubin oxidase from *Myrothecium verrucaria* (BOD) was obtained from Amano Enzyme, Japan, and 2,2′-Azino-bis(3-ethylbenzothiazoline-6-sulfonic acid) (ABTS) and 2-aminophenol (2-AP) were purchased from Tokyo Chemical Industry Co., Ltd., Japan. All chemicals were of analytical grade.

### Fabrication of EC-SAXS Cell Components

**Figure 1:**
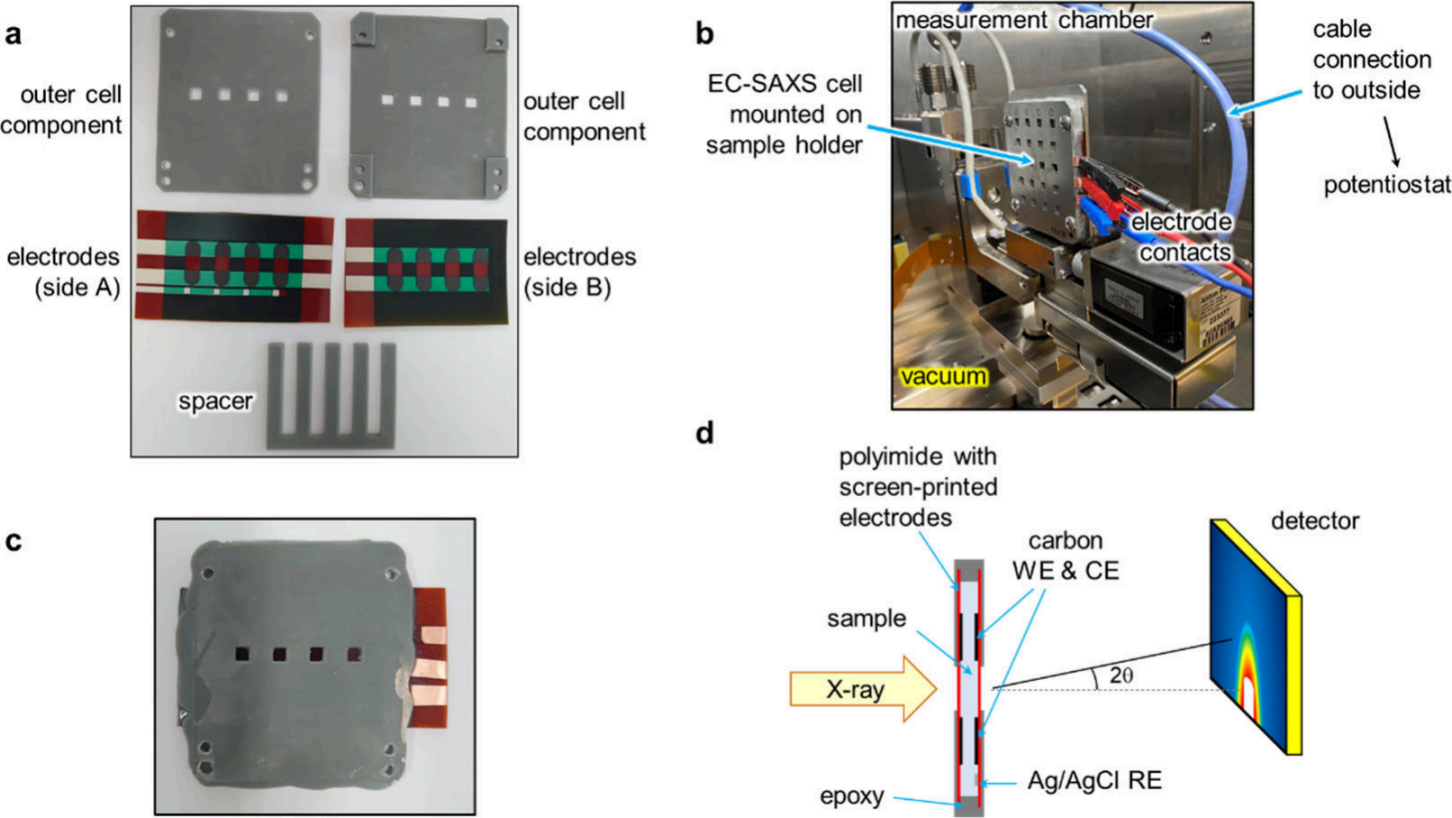
EC-SAXS cell and measurement setup. **a** Photograph of cell components: polyimide films with screen-printed electrodes, a comb-shaped epoxy resin spacer (thickness 1.5 mm), and front and back epoxy resin parts with windows for X-rays and holes for mounting on the sample holder. **b** Photograph of the EC-SAXS cell setup in the measurement chamber connected to a potentiostat. **c** Photograph of the assembled EC-SAXS cell. **d** Schematic showing a cross-section of the EC-SAXS cell during measurement.

Electrodes for the EC-SAXS cell (Figure 1a, Supplementary Figure S1) were screen-printed on a polyimide (PI) film using an LS-150TV screen printer (Newlong Seimitsu Kogyo Co. Ltd., Japan). First, electrode leads were fabricated by printing a layer of silver ink and drying at 130 °C for 15 min thrice. Next, reference electrodes were printed using Ag/AgCl ink and dried at 130 °C for 15 min. The Ag/AgCl ink was prepared by thoroughly mixing AgCl powder with a 10-fold amount of Ag ink. Then, working and counter electrodes were added by printing a layer of carbon ink and drying at 120 °C for 30 min five times. Finally, a resist layer was printed and dried at 180 °C for 1 h.

Templates for molds for EC-SAXS cell epoxy components were fabricated using a Form 3+ 3D printer (i-Maker, Japan). Molds were produced by pouring PDMS (base mixed with curing agent) over the templates and curing at 60 °C for 12 h. Cell components were created by filling the molds with epoxy resin adhesive mix and curing.

### Fabrication of EC-SAXS Cells

EC-SAXS cells were fabricated by carefully gluing the components together using epoxy resin, with the comb-shaped epoxy spacer in the middle, flanked by the two PI films with printed-on electrodes, and sandwiched by the two epoxy components (Figure 1a). During this process, the electrodes on the two PI films were connected with conducting tape, and all noncell-chamber spaces were neatly filled with epoxy resin. Holes for filling the cell chambers were left open. The connection pads for the electrodes were enhanced with conducting tape to increase connection stability. After filling the cell chamber with their respective sample solution, the cell chambers were sealed with epoxy resin. Sealed EC-SAXS cells were left to harden and settle overnight at 4 °C before EC-SAXS measurement.

### EC-SAXS Measurement of Electrochemically Oxidized and Reduced BOD

For the EC-SAXS measurements, a 10-, 20-, or 50 mg/mL solution of BOD in 100 mM phosphate buffer, pH 8.0, containing 10 mM ABTS and 10 mM 2-AP was prepared in a glovebox under an Ar atmosphere (Supplementary Table S3). A solution of 100 mM phosphate buffer, pH 8.0, containing 10 mM ABTS and 10 mM 2-AP was used as a blank. The sample and blank were sealed into an EC-SAXS cell under Ar.

EC-SAXS data were acquired using the laboratory-based SAXS system SAXSpoint 5.0 (Anton Paar GmbH, Austria) with a Cu K-α X-ray source. The detector was set at a distance of 600 mm, and data was collected over 600 s and reduced to a 1D scattering profile using the system software (Supplementary Table S3).

The EC-SAXS cell was connected to an EmStat4S potentiostat (PalmSens BV, The Netherlands) via a LAN port in the SAXSpoint 5.0 sample chamber using a modified LAN cable. A potential of −0.1 V vs Ag/AgCl was applied for over 30 min to reduce the sample. During this period, the cell was manually inverted multiple times to ensure even distribution of oxidized and reduced species within the cell. Toward the end of the 30 min interval, the cell was positioned on the sample stage, and the chamber was evacuated. Subsequently, SAXS data for the reduced sample were collected while maintaining the applied potential. Similarly, a potential of 0.8 V vs Ag/AgCl was applied for over 30 min to oxidize the sample, followed by SAXS data collection for the oxidized sample under the same conditions. During this step, the chamber was vented, the cell was inverted manually several times, and the chamber was re-evacuated. SAXS data for oxidized and reduced BOD were obtained from the same sample.

### SAXS Measurement of Chemically Oxidized and Reduced BOD

A 10, 20, or 50 mg/mL solution of BOD in 10 mM phosphate buffer, pH 7.0, prepared in an air atmosphere was used as a chemically oxidized BOD sample (Supplementary Table S2). The sample was sealed into a glass capillary (outer diameter 1.5 mm, wall thickness 0.01 mm) using Bond Quick 5 epoxy resin adhesive (Konishi Co., Ltd., Japan). The blank for chemically oxidized BOD consisted of 10 mM phosphate buffer, pH 7.0, sealed into a capillary in air. For the chemically reduced BOD sample, a solution of 10-, 20-, or 50 mg/mL BOD in 10 mM phosphate buffer, pH 7.0, containing 50 mM ABTS was prepared under Ar (Supplementary Table S2). The chemically reduced BOD sample was sealed into a capillary under Ar. The blank for chemically reduced BOD was 10 mM phosphate buffer, pH 7.0, containing 50 mM ABTS sealed into a capillary under Ar. Sealed capillaries were stored overnight at 4 °C before measurement.

SAXS data for samples sealed into capillaries were acquired with the laboratory-based SAXS system Xeuss 3.0 (Xenocs SAS, France) with a Cu Kα X-ray source. The detector was set at a distance of 500 mm, and data was collected over 300 s and reduced to a 1D scattering profile using the system software (Supplementary Table S3).

### SAXS Analysis

SAXS data were analyzed using the ATSAS software package (Supplementary Table S4).^40^ Blanks were subtracted using the processing tools in the PRIMUS program.^40^ The distance distribution function and the maximal particle dimension *D*_max_ were determined using the analysis tool in the program. *Ab initio* modeling was conducted ten times with DAMMIF^41^ and the results were averaged using DAMAVER.^42^ Refinement modeling was carried out with SREFLEX^44^ and CRYSOL,^43^ using the crystal structure of BOD from *M. verrucaria* (PDB: 6IQZ).^19^ The *Ab initio* and refined models were superimposed using CIFSUP,^40^ and 3D structures were visualized using PyMOL (The PyMOL Molecular Graphics System, version 2.5.2 Schrödinger, LLC).

## Results and Discussion

### Design of the EC-SAXS Cell

The EC-SAXS cell was specifically designed to fit into the sample holder for solids in the SAXSpoint 5.0 laboratory-based SAXS system (Figure 1). We selected disposable materials and fabrication methods to create a nonreversible tight seal that can be easily sealed within a glovebox. The cell comprises four chambers, each capable of holding a different sample. Each chamber features a 5 × 5 mm window for X-rays. The X-ray path penetrates a PI film, the sample solution (1.5 mm length), and another PI film, with electrodes positioned above and below this path. These window positions match those in the middle row of the sample holder. The design, materials, and manufacturing process were optimized to ensure the EC-SAXS cell maintained integrity under the vacuum conditions of the measuring chamber. As part of the optimization, cells were tested for leakage—both externally and between chambers. For the latter, SAXS data from cells with all chambers filled with identical contents were compared to data from cells with chambers containing different contents, such as sample, blank, or empty. Furthermore, the reliability of EC-SAXS cell fabrication was verified by comparing SAXS measurements of identically prepared samples in different cells (Supplementary Figure 5a).

### Scattering Profiles of Oxidized and Reduced BOD

High-redox-potential mediators required to oxidize BOD in spectroelectrochemical experiments are not commercially available and necessitate the use of cyanides for their synthesis.^62,63^ Consequently, this study explored electrochemically oxidized and reduced BOD at pH 8.0, where the redox potential of BOD is known to decrease with increasing pH,^65^ being 0.34 V vs Ag/AgCl/sat. KCl.^65^ The mediators ABTS and 2-AP were chosen for their redox potentials, approximately 0.1 V higher and 0.2 V lower than that of BOD65, respectively, making them suitable for this study’s oxidation and reduction experiments. Scattering profiles of mediators without BOD (background) and a simple reaction scheme are depicted in Supplementary Figures S5b and S6.

**Figure 2:**
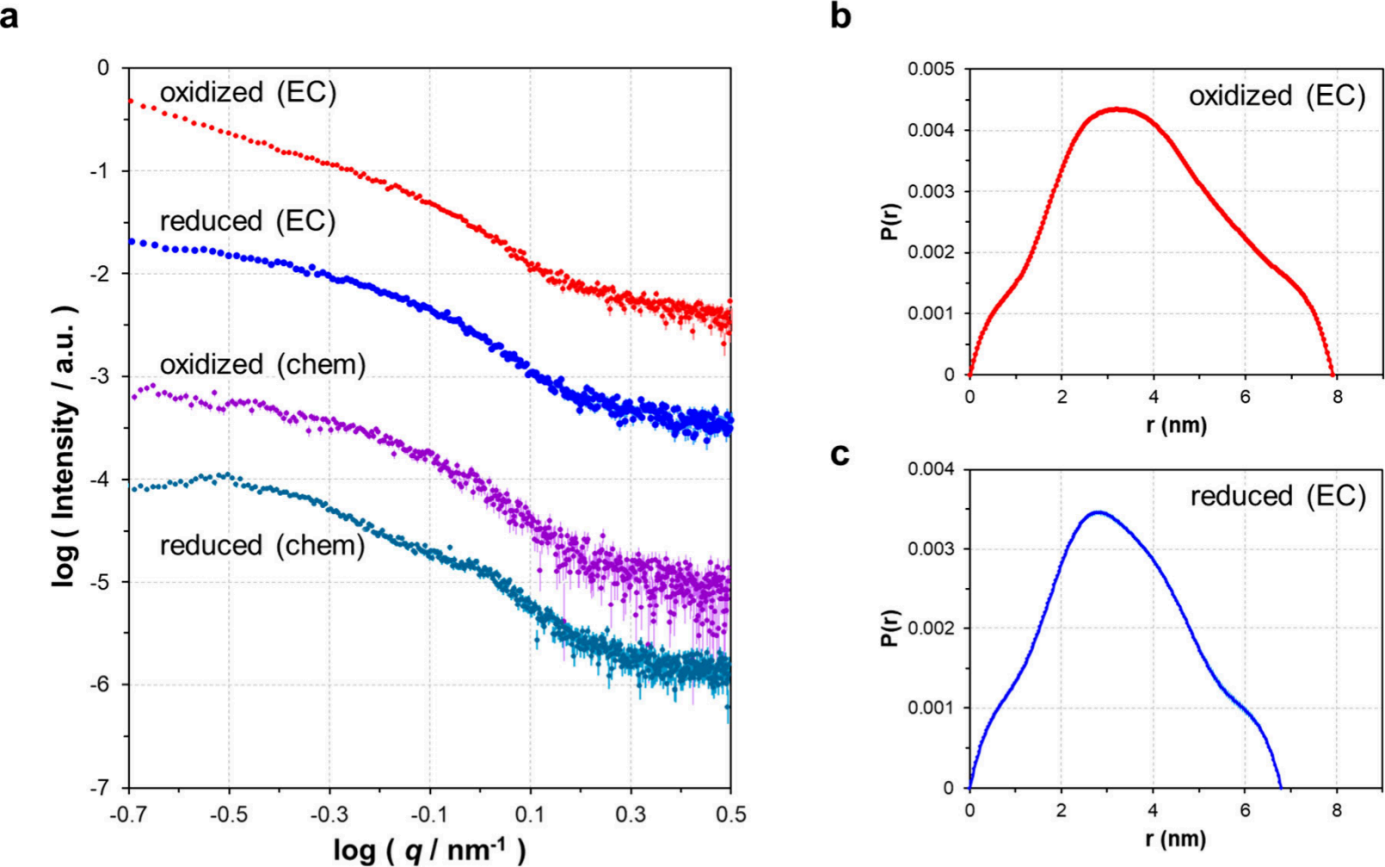
SAXS intensity data and distance distribution for oxidized and reduced BOD (50 mg/mL). **a** Scattering intensity for electrochemically (EC) and chemically (chem) oxidized and reduced BOD, presented in a double-logarithmic plot. Scattering profiles were shifted along the intensity axis to enhance visibility. **b** Distance distribution function P(r) for electrochemically oxidized BOD at 0.8 V vs Ag/AgCl. **c** Distance distribution P(r) for electrochemically reduced BOD at −0.1 V vs Ag/AgCl.

SAXS data were acquired at −0.1 and 0.8 V vs Ag/AgCl, corresponding to the reduced and oxidized states of BOD, respectively (Figure 2; Supplementary Figures S7, S8, and S9). Here, *q* is the momentum transfer variable, defined as *q* = (4π sin θ)/λ, where 2θ is the scattering angle, and λ the incident X-ray wavelength. The intensity of SAXS signals for both electrochemically oxidized and reduced BOD decreased with decreasing BOD concentration (Supplementary Figures S7a,b and S8a,b). Clear differences in the scattering profiles of oxidized and reduced BOD at the same concentration were observed (Figure 2a, Supplementary Figure S9a), and these differences were reproducible (Supplementary Figure S9a).

These differences were reproducible (Supplementary Figure S9a). The distance distribution functions derived from these scattering data suggested that reduced BOD had a more compact shape than oxidized BOD (Figure 2b,c). Electrochemically reduced BOD had an average maximal particle dimension *D*_max_ of 6.7 ± 0.2 nm, while that for oxidized BOD was 7.5 ± 0.4 nm (Supplementary Tables S12 and S13). The molecular weight determined by SAXS was 58 ± 2 kDa and 63 ± 9 kDa (Supplementary Tables S12 and S13) for electrochemically reduced and oxidized BOD, respectively, within error margins of each other and within ±15% of the value reported in literature (66 kDa^66^).

For comparison, SAXS data were also acquired for chemically oxidized and reduced BOD (Figure 2, Supplementary Figures S7, S8, S10, S11). These data were obtained at pH 7.0, a pH commonly used for BOD characterization, especially in studies involving BOD electrochemistry. At this pH, ABTS serves as a reducing agent, and BOD was chemically reduced by adding ABTS. BOD was also oxidized by molecular oxygen in an air-saturated environment. Despite differences in sample preparation, the variation between the scattering profiles of chemically oxidized and reduced BOD showed similar trends to those observed with electrochemically treated BOD (Figure 2a, Supplementary Figure S10). The concentration dependency in chemically treated samples was much less pronounced (Supplementary Figures S7c,d and S8c,d), possibly due to differences in instruments and measurement parameters (Supplementary Table S3). The average maximum particle dimension *D*_max_ for chemically reduced BOD was measured at 6.7 ± 0.1 nm, while for oxidized BOD, it was 7.5 ± 0.5 nm (Supplementary Figure S11; Supplementary Tables S14 and S15).

These findings demonstrate that reduced BOD consistently maintains a more compact shape than oxidized BOD, regardless of electrochemical or chemical oxidation/reduction and other conditions, such as pH.

### *Ab initio* Models of Oxidized and Reduced BOD

**Figure 3:**
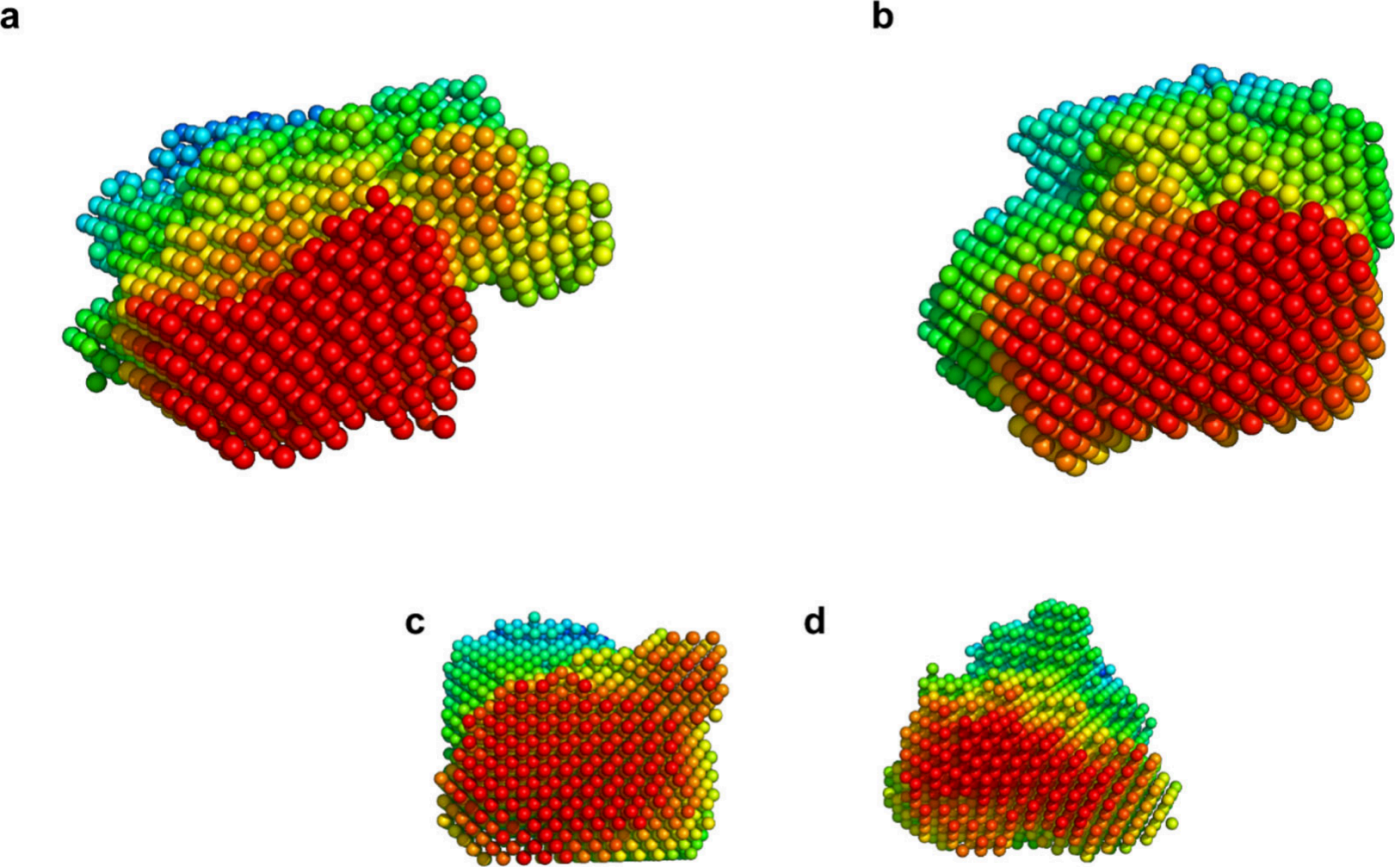
*Ab initio* models of oxidized and reduced BOD. (Damaver) The beads are color-coded to indicate spatial positioning, with red beads in front and green/blue beads in the background; 50 mg/mL BOD samples. **a** Electrochemically oxidized BOD. *D*_max_ = 7.82 nm. **b** Electrochemically reduced BOD. *D*_max_ = 6.80 nm. **c** Chemically oxidized BOD. *D*_max_ = 7.49 nm. **d** Chemically reduced BOD. *D*_max_ = 6.73 nm.

Ab initio models for both oxidized and reduced BOD were developed (Figure 3^41^) using DAMMIF and^42^ DAMAVER. Briefly, these software tools begin with a generic model of densely packed particle and solvent beads. Random beads are reassigned as solvent or particle, and the calculated scattering profiles before and after reassignment are compared and evaluated against the experimental data. Less fitting models are discarded, and the process is repeated until a good fit is achieved. Finally, multiple generated models are averaged to produce the most likely bead model.

Models derived from EC-SAXS and standard SAXS data were similar, particularly in the differences between oxidized and reduced BOD obtained using either method. Reduced BOD appeared slightly more compact than oxidized BOD, though this observation was not statistically significant. Nevertheless, this trend aligns with the smaller size inferred for reduced BOD from the distance distribution function (Supplementary Tables S12–S15).

### Refined High-Resolution Models of Oxidized and Reduced BOD

**Figure 4:**
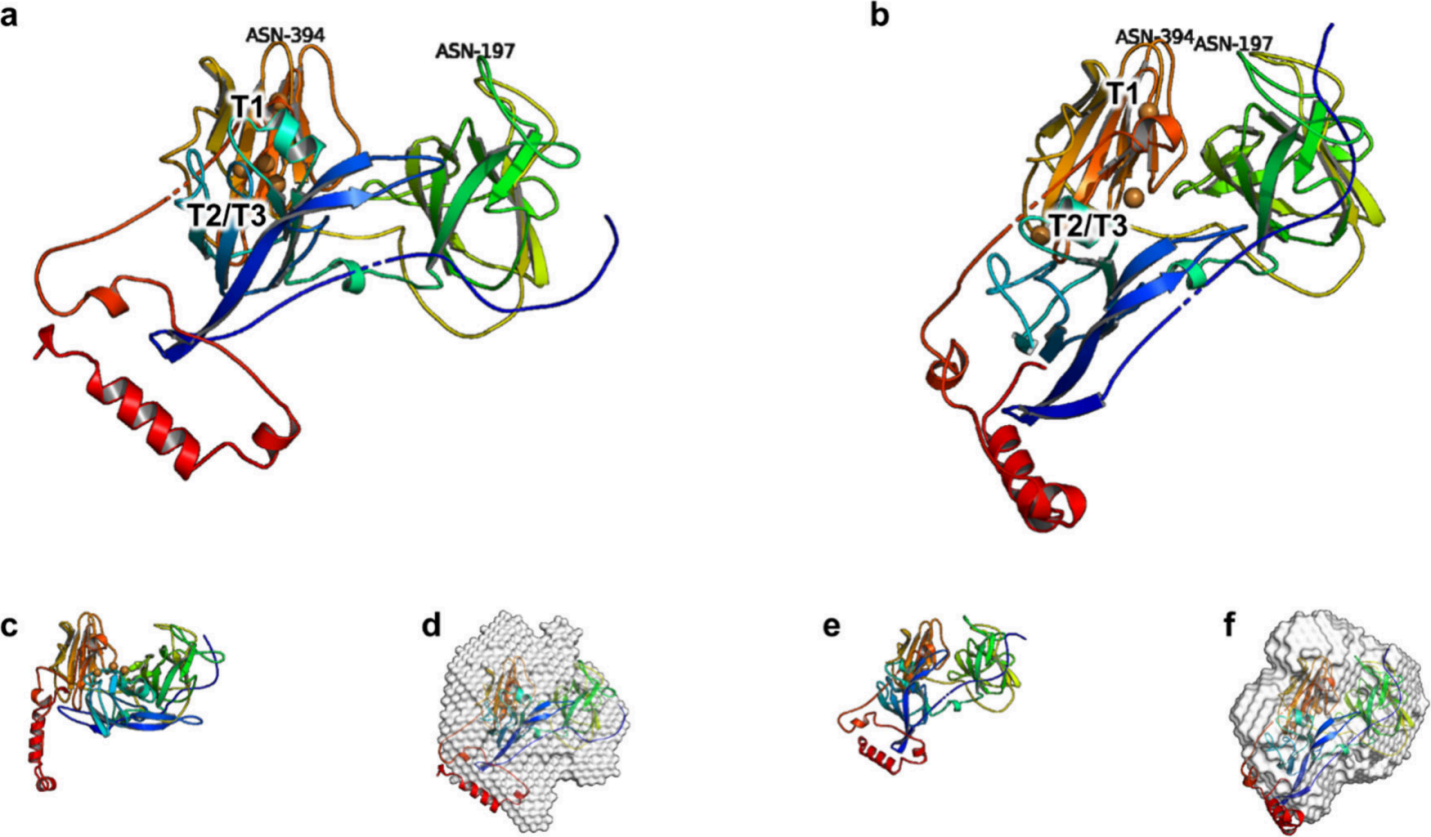
Refined high-resolution models of oxidized and reduced BOD. Models are derived from PDB entry 6IQZ;^19^ 50 mg/mL BOD samples. **a** Electrochemically oxidized BOD with an open structure. T1 Cu, T2/T3 site, Asn-197, and Asn-394 are marked. **b** Electrochemically reduced BOD with a closed structure. T1 Cu, T2/T3 site, Asn-197, and Asn-394 are marked. **c** Chemically oxidized BOD with an open structure. **d** Electrochemically oxidized BOD with the *ab initio* model as an envelope. **e** Chemically reduced BOD with a closed structure. **f** Electrochemically reduced BOD with the *ab initio* model as an envelope.

High-resolution models of oxidized and reduced BOD were refined from the existing crystal structure of BOD (PDB entry 6IQZ)^19^ using corresponding SAXS data (Figure 4; Supplementary Figures S16–S17). Refinement was conducted using SREFLEX^44^ and^43^ CRYSOL. Briefly, these software tools start with a known high-resolution model and generate numerous modified structures by slightly altering random parts of the initial structure. Predicted scattering profiles are compared against experimental data, and less fitting models are discarded. The process is iterated until a good fit is achieved, yielding nine high-resolution models that better match the experimental conditions. Additionally, solvent densities were set to 0.33 e/Å^3^ for standard SAXS and 0.35 e/Å^3^ for EC-SAXS due to differences in ionic strength.

These models revealed that BOD can exhibit either an open or closed structure. The open structure features a more accessible T1 Cu site. While either type was found among the conformers of oxidized and reduced BOD, oxidized BOD showed a predominantly open structure, and reduced BOD was predominantly closed (Supplementary Figures S16–S17, Supplementary Videos V1–V4). This difference is reflected in the mean distance between residues Asn-197 and Asn-394, which is longer in the open structure than that in the closed one. The mean distances for electrochemically oxidized and reduced BOD were measured at 2.8 ± 0.7 nm and 1.7 ± 0.6 nm, respectively. The difference was less pronounced for chemically oxidized and reduced BOD (3.0 ± 0.9 nm and 2.5 ± 1 nm, respectively).

### Open and Closed Structure of BOD

In oxidized BOD, the copper ions, including T1 Cu, exhibit strong positive charges as Cu^2+^. Conversely, in reduced BOD, the copper ions (Cu+) carry weaker charges. It is plausible that the open structure of oxidized BOD arises from the repulsion of positively charged amino acid residues or the attraction of negatively charged environmental molecules, which may penetrate and expand the protein structure. Given that the relevant BOD sequence contains a balanced proportion of positively and negatively charged residues, the latter scenario seems more likely. This hypothesis is further supported by the more pronounced structural differences between oxidized and reduced BOD observed in EC-SAXS analysis, where a phosphate buffer ten times more concentrated was used, increasing the abundance of negatively charged molecules. In the absence of such molecules, BOD may fluctuate randomly between open and closed structures regardless of redox state. Negatively charged molecules likely stabilize the open structure in oxidized BOD or the closed structure in reduced BOD. Further investigation is needed to substantiate this hypothesis. Notably, the natural substrate for oxidized BOD, bilirubin, which contains two carboxyl groups and is negatively charged, requires access to T1 Cu for reactivity—a process facilitated by the open structure. Conversely, molecular oxygen, the natural substrate for reduced BOD, is small, uncharged, and readily accesses the reaction site (T2/T3) in the closed structure.

These findings highlight the utility of EC-SAXS in potential-dependent structural analysis of redox enzymes and redox-active proteins. This novel approach promises to remarkably advance our understanding of the reaction mechanisms in these redox enzymes and redox-active proteins. Furthermore, insights into structural changes during the redox reaction could enhance the development of tailor-made immobilization strategies, thereby improving the performance of biosensors, biofuel cells, and other bioelectronics.

## Conclusions

In this study, a novel method for the investigation of reduced and oxidized redox-active proteins was introduced in the form of EC-SAXS. EC-SAXS combines electrochemical and small-angle X-ray characterization. Using this method, it was demonstrated that a small redox-enzymes such as BOD can be very flexible during its redox cycle. BOD alternates between an open and a close structure. The open structure is predominantly favored by the oxidized enzyme, whereas the closed structure is preferred by the reduced state. EC-SAXS represents a promising novel method for advancing our understanding of the reaction mechanisms of redox enzymes. Unlike standard SAXS, EC-SAXS allows the analysis of oxidized and reduced enzymes in the presence of identical additives. Consequently, differences observed in EC-SAXS data can be attributed more reliably to the redox state of the sample, eliminating the need to account for variations in additives. Ultimately, a deeper understanding of the redox mechanisms of enzymes will facilitate the development of advanced, tailor-made immobilization strategies, thereby enhancing the performance of enzyme-based biodevices.

## Supporting Information Available

Video V1 – Electrochemically oxidized BOD; refined high-resolution model switching through nine conformers and back, superimposed with *ab initio* model envelope (MPG)
Video V2 – Electrochemically reduced BOD; refined high-resolution model switching through nine conformers and back, superimposed with *ab initio* model envelope (MPG)
Video V3 – Chemically oxidized BOD; refined high-resolution model switching through nine conformers and back (MPG)
Video V4 – Chemically reduced BOD; refined high-resolution model switching through nine conformers and back (MPG)
Figure S1 – Schematic of printing layers of EC-SAXS cell electrodes; Table S2 – Sample preparation; Table S3 – SAXS measurement parameters; Table S4 – SAXS analysis software; Figure S5 – Reproducibility and background scattering of EC-SAXS cell; Figure S6 – Reaction scheme; Figure S7 – Double-logarithmic plots of SAXS scattering intensity data; Figure S8 – Logarithmic plots of SAXS scattering intensity data; Figures S9 and S10 – Further representations of EC-SAXS scattering data; Figure S11 – Distance distribution for chemically oxidized and reduced BOD; Tables S12–S15 – Determined size parameters; Figures S16 and S17 – Refined high-resolution model of BOD (PDF)

## Supplementary Material

la4c03661_si_001.mpg

la4c03661_si_002.mpg

la4c03661_si_003.mpg

la4c03661_si_004.mpg
