# Supplementary material for: Electrochemical Small-Angle X-ray Scattering for Potential-Dependent Structural Analysis of Redox Enzymes
Source: Langmuir. 2024 Dec 31;41(1):383–91. doi: 10.1021/acs.langmuir.4c03661 (PMC11736843; doi:10.1021/acs.langmuir.4c03661)
Supplement: Supplementary file 5 — la4c03661_si_006.pdf [file la4c03661_si_006.pdf]

Supplementary Information

Electrochemical Small-angle X-Ray Scattering for Potential-dependent Structural Analysis of Redox Enzymes

Noya Loew[1], Chika Miura[1], Chiaki Sawahara[1], Saki Otobe[2], Taku Ogura[2,3], Yuichi Takasaki[4], Hikari Watanabe[1], Isao Shitanda[1,3,*], Masayuki Itagaki[1,3]

[1] Department of Pure and Applied Chemistry, Faculty of Science and Technology, Tokyo University of Science, 2641 Yamazaki, Noda, Chiba 278-8510, Japan

[2] Nikko Chemicals Co. Ltd., 3-24-3 Hasune, Itabashi-ku, Tokyo 174-0046, Japan

[3] Research Institute for Science and Technology, Tokyo University of Science, 2641 Yamazaki, Noda, Chiba 278-8510, Japan

[4] Anton Paar Japan K.K., Riverside Sumida 1F, 1-19-9, Tsutsumi-dori, Sumida-ku, Tokyo, 131-0034, Japan

[*] **Corresponding author**: Isao Shitanda

Department of Pure and Applied Chemistry, Faculty of Science and Technology, Tokyo University of Science, 2641 Yamazaki, Noda, Chiba 278-8510, Japan

E-mail: shitanda@rs.tus.ac.jp

Telephone: +81-4-7124-1501, Fax: +81-4-7123-9890

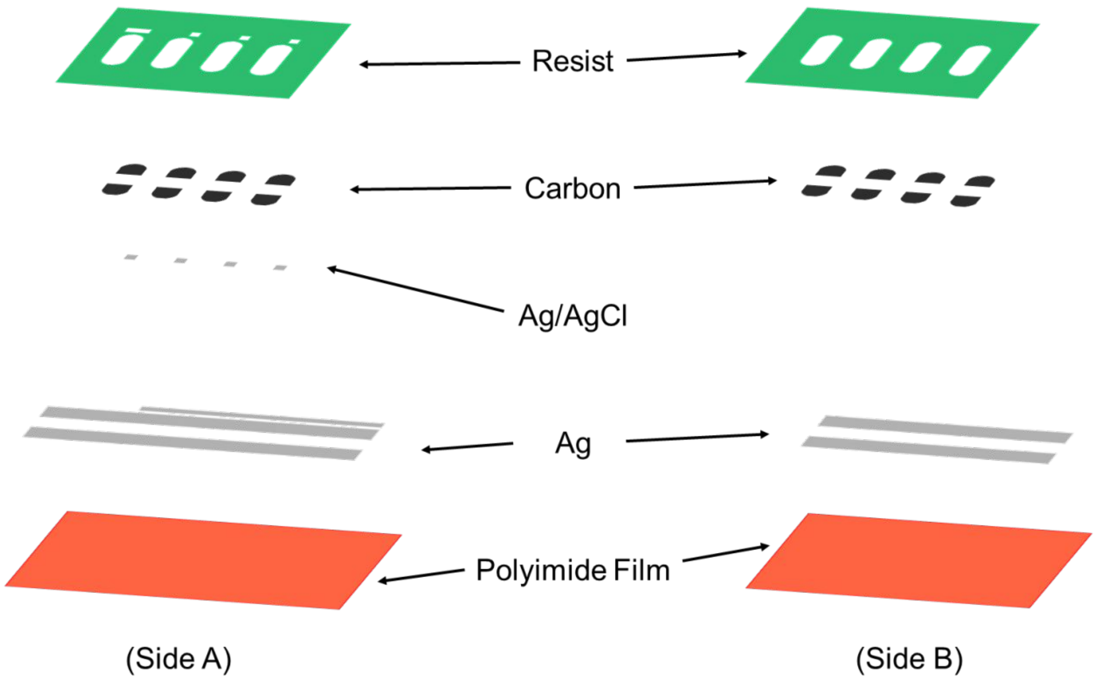

Supplementary Figure S1. Schematic of printing layers of EC-SAXS cell electrodes. From bottom to top: polyimide film (printing substrate), Ag ink (3 layers), Ag/AgCl ink (1 layer, only Side A electrodes), carbon ink (5 layers), and resist ink (1 layer).

Supplementary Table S2. Sample preparation.

	EC-SAXS	SAXS (Chem ox*)	SAXS (Chem red*)
Protein	Bilirubin oxidase from *Myrothecium verrucaria*		
Source	Amano Enzyme (Amano-3)		
Molecular weight	66 kDa[1]		
Concentration (mg/mL)	50, 20, or 10	50, 20, or 10	50, 20, or 10
Solvent	100 mM phosphate buffer, pH 8.0	10 mM phosphate buffer, pH 7.0	
Additives	10 mM ABTS, 10 mM 2-AP	none	50 mM ABTS
Ar purging/O_2 presence	Ar	O_2	Ar

* Chem ox: chemically oxidized; Chem red: chemically reduced.

Supplementary Table S3. SAXS measurement parameters.

	EC-SAXS	SAXS
Instrument	SAXSpoint 5.0	Xeuss 3.0
Data processing	SAXSdrive ver 3.01	specfe-xenocs ver 2.1.4.10

Wavelength	1.5406 Å	1.5406 Å
Beam size	3 mm	
Camera distance	600 mm	500 mm
Exposure time	600 s	300 s
Sample cell	EC-SAXS cell	capillary

Supplementary Table S4. SAXS analysis software.

SAXS data reduction	SAXSanalysis ver 4.20; specfe-xenocs ver 2.1.4.10
Background substraction	ATSAS ver 3.0.4
Distance distribution	GNOM ver 5.0
Bead modeling	DAMMIF; DAMAVER 5.0
Refinement modeling	SREFLEX ATSAS 3.0.4
Graphical visualization	PyMOL 2.5.2

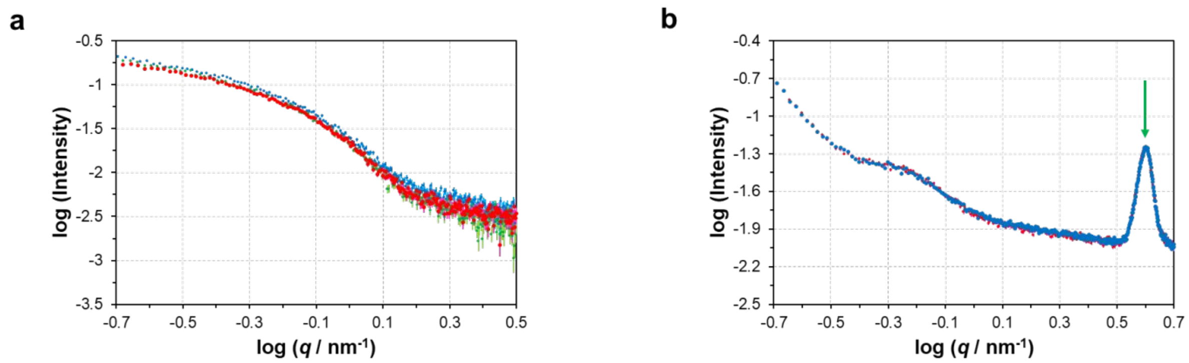

Supplementary Figure S5. Reproducibility and background scattering of the EC-SAXS cell. **(a)** Scattering profiles of three independent 50 mg/mL BOD samples before the application of potential using the EC-SAXS cell. **(b)** Background SAXS scattering profiles of oxidized (red) and reduced (blue) mediators in 100 mM potassium phosphate buffer. The green arrow indicates a peak attributed to polyimide.

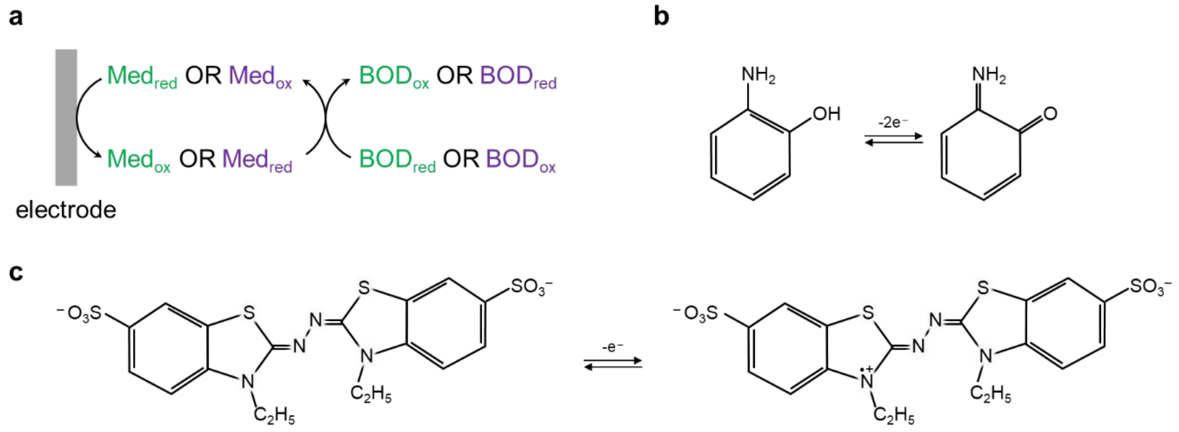

Supplementary Figure S6. Reaction scheme. Reaction scheme.

(a) Schematic representation of the mediated oxidation (green) or reduction (violet) of BOD.

(b) Redox reaction of 2-AP. **(c)** Redox reaction of ABTS.

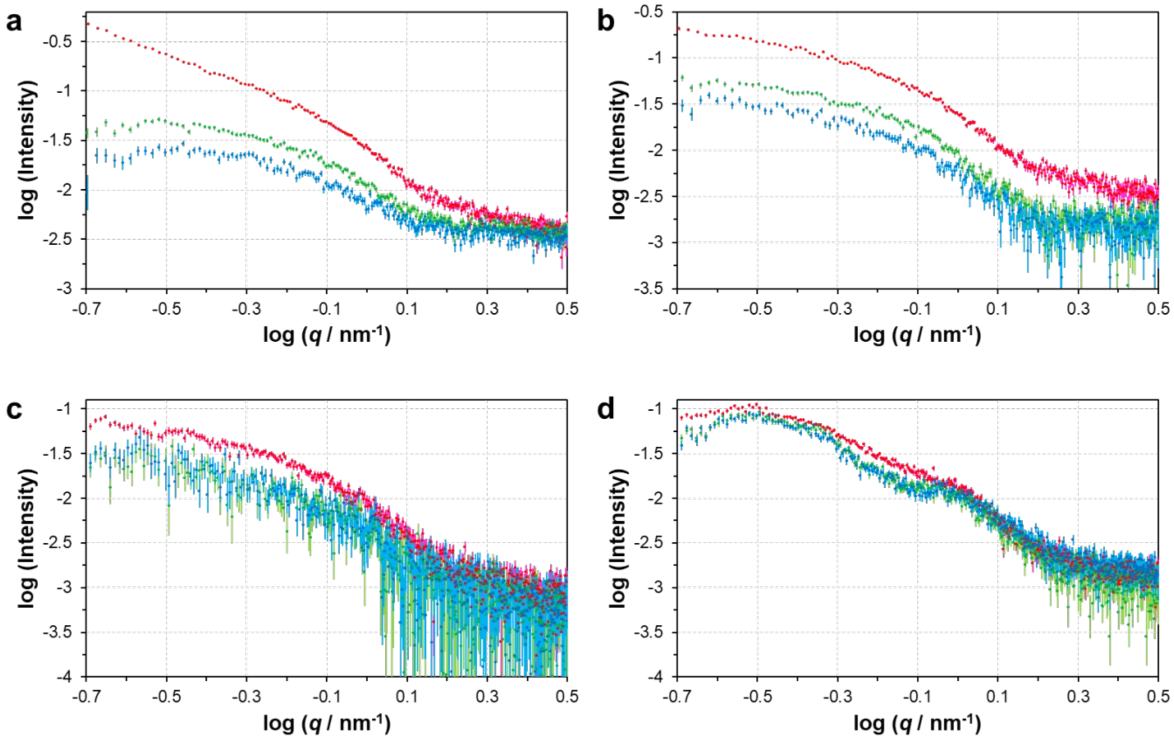

Supplementary Figure S7. Double logarithmic plots of SAXS scattering intensity data.

Oxidized and reduced BOD. Various concentrations: red – 50 mg/mL; green – 20 mg/mL; blue – 10 mg/mL. **a** Electrochemically oxidized BOD. 0.8 V vs. Ag/AgCl. **b** Electrochemically reduced BOD. -0.1V vs. Ag/AgCl. **c** Chemically oxidized BOD. Presence of O_2. **d** Chemically reduced BOD. Presence of ABTS.

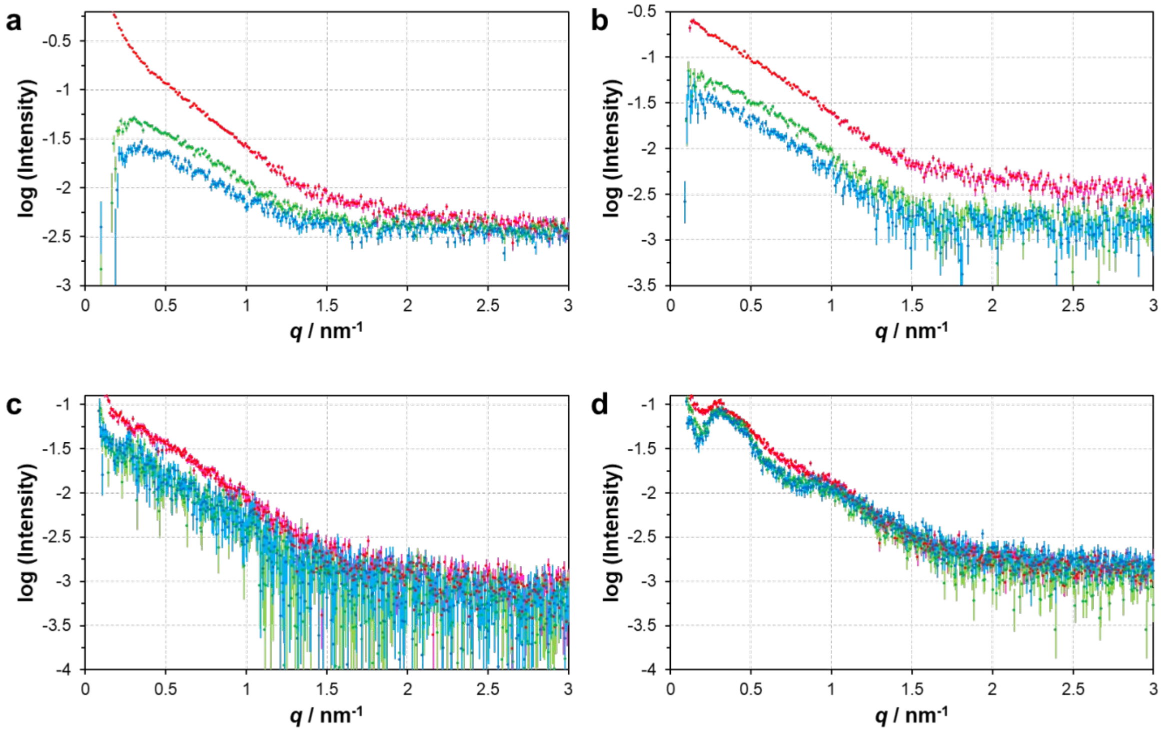

Supplementary Figure S8. Logarithmic plots of SAXS scattering intensity data. Oxidized and reduced BOD. Various concentrations: red – 50 mg/mL; green – 20 mg/mL; blue – 10 mg/mL. **a** Electrochemically oxidized BOD. 0.8 V vs. Ag/AgCl. **b** Electrochemically reduced BOD. -0.1V vs. Ag/AgCl. **c** Chemically oxidized BOD. Presence of O_2. **d** Chemically reduced BOD. Presence of ABTS.

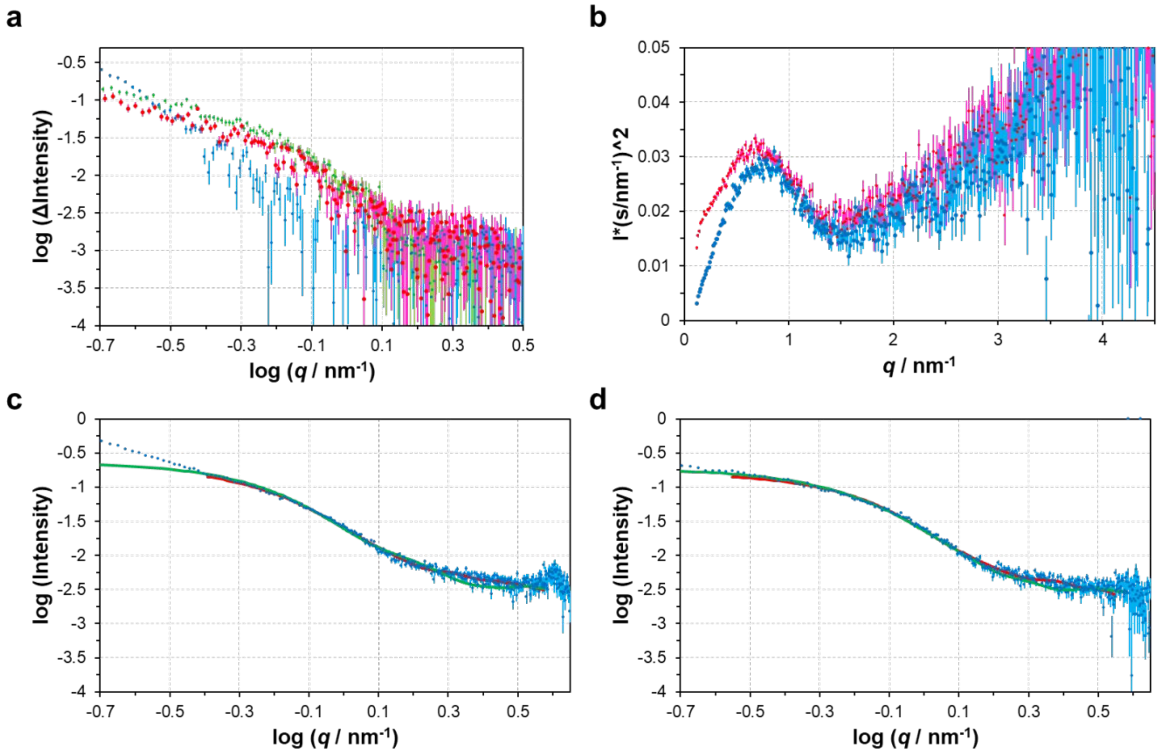

Supplementary Figure S9. Further representations of EC-SAXS scattering data of electrochemically oxidized and reduced BOD (50 mg/mL). **(a)** Double-logarithmic plot of the intensity difference between oxidized and reduced BOD ($N = 3$). **(b)** Kratky plot. Red: oxidized BOD, Blue: reduced BOD. **(c)** Double-logarithmic plot of oxidized BOD. Blue: experimental data, Red: predicted data of the representative *ab initio* model, Green: predicted data of the representative refined high-resolution model. **(d)** Double-logarithmic plot of reduced BOD. Blue: experimental data, Red: predicted data of the representative *ab initio* model, Green: predicted data of the representative refined high-resolution model.

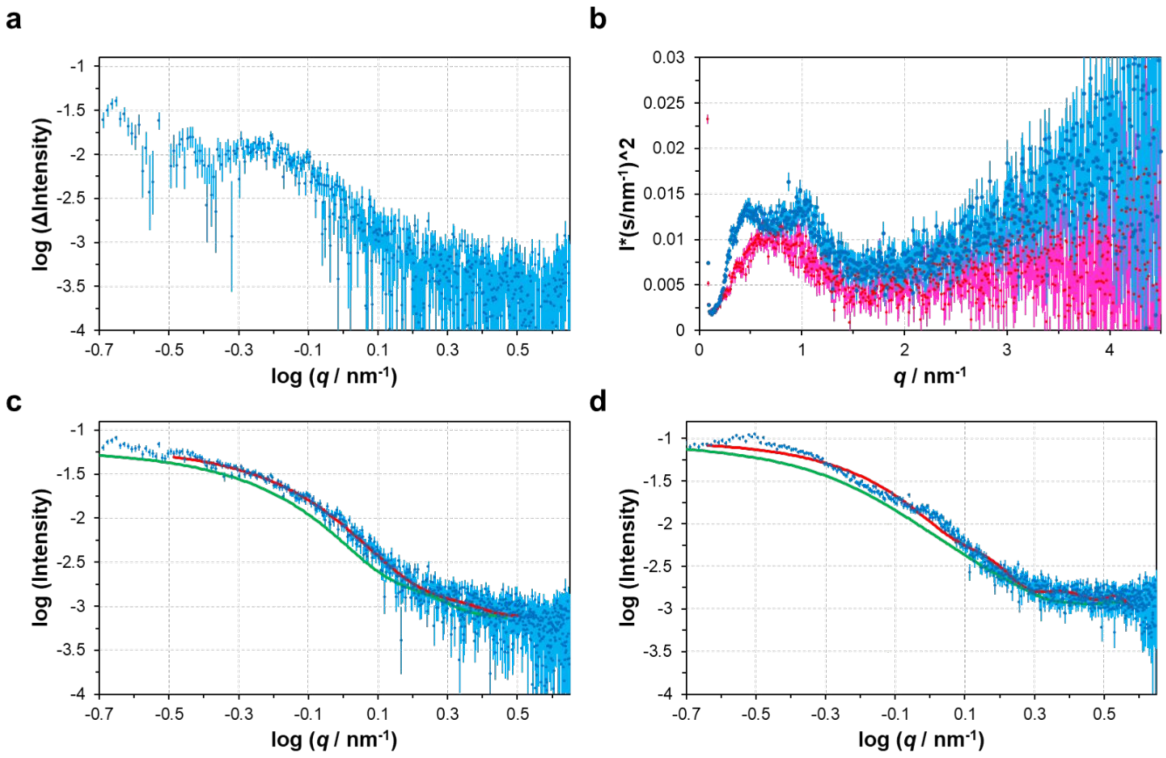

Supplementary Figure S10. Further representations of SAXS scattering data of chemically oxidized and reduced BOD (50 mg/mL). **(a)** Double-logarithmic plot of the intensity difference between oxidized and reduced BOD. **(b)** Kratky plot. Red: oxidized BOD, Blue: reduced BOD. **(c)** Double-logarithmic plot of oxidized BOD. Blue: experimental data, Red: predicted data of the representative *ab initio* model, Green: predicted data of the representative refined high-resolution model. **(d)** Double-logarithmic plot of reduced BOD. Blue: experimental data, Red: predicted data of the representative *ab initio* model, Green: predicted data of the representative refined high-resolution model.

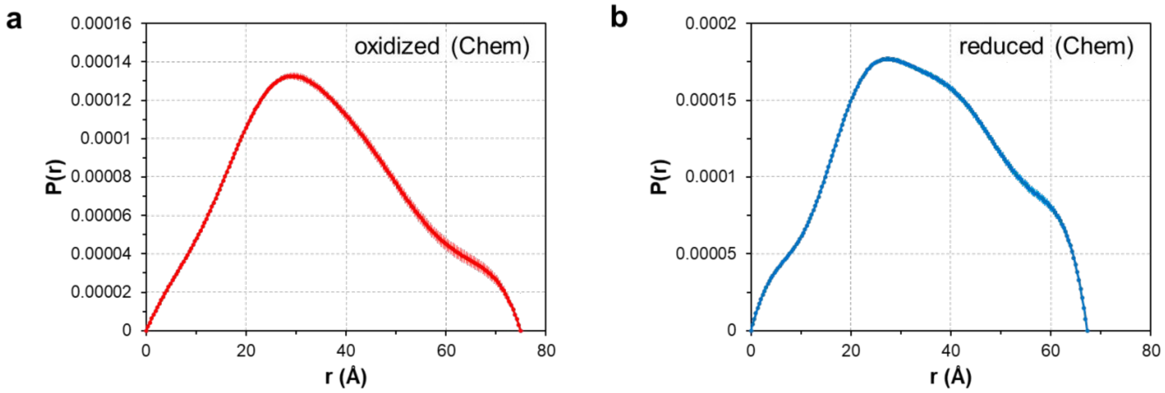

Supplementary Figure S11. Distance distribution for chemically oxidized and reduced BOD (50 mg/mL). **a** Distance distribution function P(r) for chemically oxidized BOD. **b** Distance distribution function P(r) for chemically reduced BOD.

Supplementary Table S12. Determined size parameters I. Electrochemically oxidized BOD.

	Electrochemically oxidized BOD			
	50 mg/mL	20 mg/mL	10 mg/mL	average
D_{max} (nm)	7.82	7.62	7.04	7.5 ± 0.4
R_g (nm)*	3.1 ± 0.1	4.8 ± 1	3.9 ± 0.8	3.9 ± 0.8
MW (kDa)	57.9	57.2	72.3	62.5 ± 9
Relative MW**	88%	87%	110%	95%
Chi² (DAMMIN)	1.447	1.400	1.337	
Chi² (DAMMIF)*1	1.450 ± 0.002	1.368 ± 0.007	1.4 ± 0.1	
Chi² (SREFLEX)*2	3.8 ± 0.5	3.6 ± 0.2	3.8 ± 0.2	

*: Radius of Gyration;**: % of 66 kDa[1]; *1: average of 10 fits; *2: average of 5 restricted and 4 unrestricted fits.

Supplementary Table S13. Determined size parameters II. Electrochemically reduced BOD.

	Electrochemically reduced BOD			
	50 mg/mL	20 mg/mL	10 mg/mL	average
D_{max} (nm)	6.80	6.46	6.72	6.7 ± 0.2
R_g (nm)*	3.7 ± 0.2	3.2 ± 0.2	4.4 ± 0.7	3.8 ± 0.6
MW (kDa)	58.9	55.4	58.8	57.7 ± 2
Relative MW**	89%	84%	89%	87%

Chi^2 (DAMMIN)	2.253	1.190	1.033
Chi^2 (DAMMIF)[*1]	2.314 ± 0.003	1.174 ± 0.002	1.031 ± 0.001
Chi^2 (SREFLEX)[*2]	2.4 ± 0.2	1.22 ± 0.02	1.17 ± 0.02

*: Radius of Gyration;**: % of 66 kDa[1]; *1: average of 10 fits; *2: average of 5 restricted and 4 unrestricted fits.

Supplementary Table S14. Determined size parameters III. Chemically oxidized BOD.

	Chemically oxidized BOD			
	50 mg/mL	20 mg/mL	10 mg/mL	average
D_{max} (nm)	7.49	7.06	8.03	7.5 ± 0.5
R_g (nm)*	3.5 ± 0.2	3.1 ± 0.3	3.4 ± 0.5	3.3 ± 0.2
MW (kDa)	65.2	58.9	68.8	64 ± 5
Relative MW**	99%	89%	104%	97%
Chi^2 (DAMMIN)	1.019	0.949	0.996	
Chi^2 (DAMMIF)*1	1.009 ± 0.001	0.958 ± 0.001	0.995 ± 0.000	
Chi^2 (SREFLEX)*2	1.29 ± 0.06	1.04 ± 0.01	1.09 ± 0.01	

*: Radius of Gyration;**: % of 66 kDa[1]; *1: average of 10 fits; *2: average of 5 restricted and 4 unrestricted fits.

Supplementary Table S15. Determined size parameters IV. Chemically reduced BOD.

	Chemically reduced BOD			
	50 mg/mL	20 mg/mL	10 mg/mL	average
D_{max} (nm)	6.73	6.71	6.55	6.7 ± 0.1
R_g (nm)*	3.2 ± 0.1	3.3 ± 0.1	3.4 ± 0.1	3.3 ± 0.2

MW (kDa)	67.3	70.3	71.1	70 ± 2
Relative MW**	102%	107%	108%	105%
Chi^2 (DAMMIN)	3.000	3.590	2.797	
Chi^2 (DAMMIF)*1	2.986 ± 0.004	3.553 ± 0.002	2.817 ± 0.002	
Chi^2 (SREFLEX)*2	2.9 ± 0.2	3.2 ± 0.1	3.7 ± 0.2	

*: Radius of Gyration;**: % of 66 kDa[1]; *1: average of 10 fits; *2: average of 5 restricted and 4 unrestricted fits.

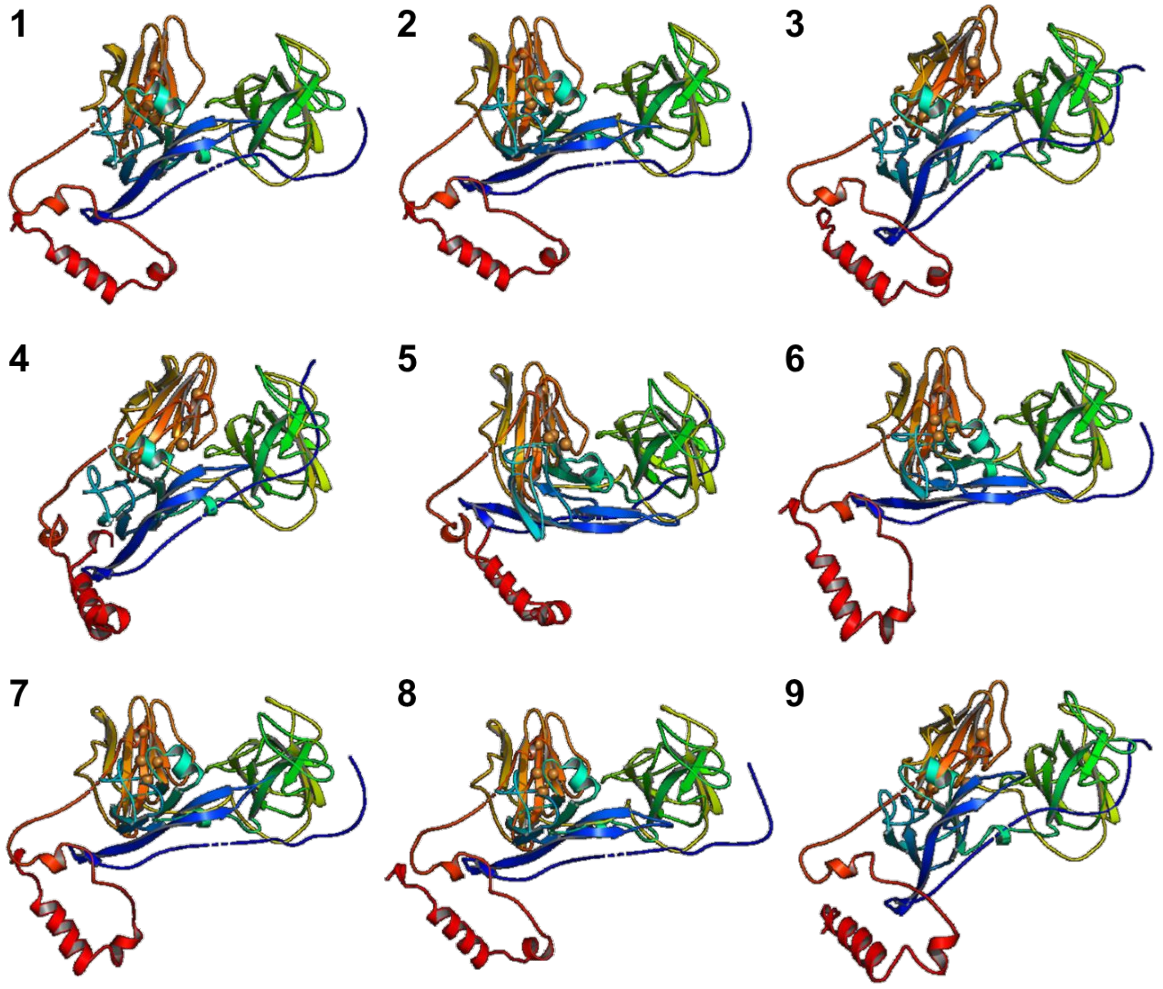

Supplementary Figure S16. Refined high-resolution model of oxidized BOD. (**1–9**) Nine conformers derived fromEC-SAXS analysis. (50 mg/mL).

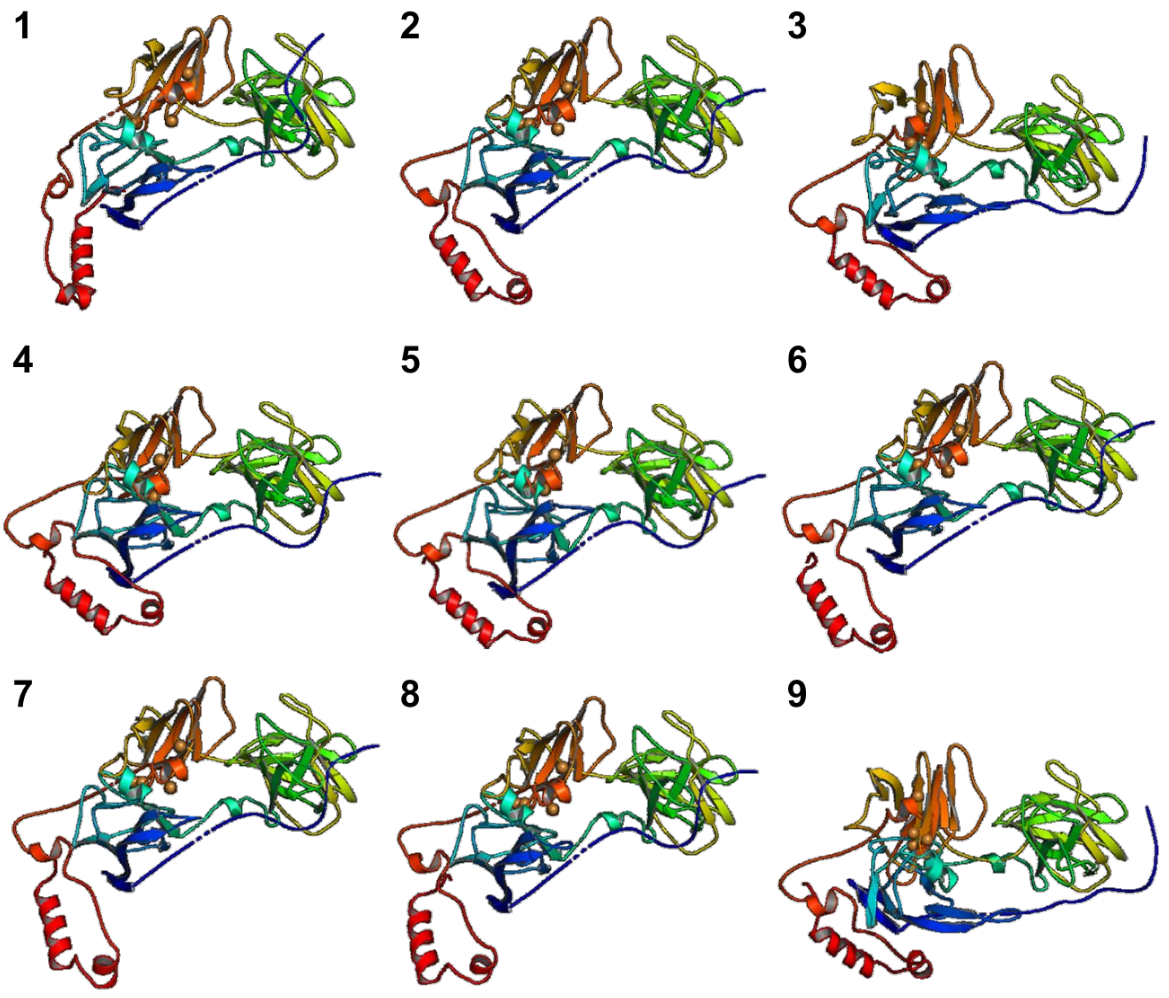

Supplementary Figure S17. Refined high-resolution model of reduced BOD. (**1–9**) Nine conformers derived from EC-SAXS analysis. (50 mg/mL).

References

1. Mano, N.; Edembe, L. Bilirubin Oxidases in Bioelectrochemistry: Features and Recent Findings. *Biosens. Bioelectron.* **2013**, *50*, 478–485. https://doi.org/10.1016/j.bios.2013.07.014.
